# Diffuse midline gliomas, H3 K27M-mutant are associated with less peritumoral edema and contrast enhancement in comparison to glioblastomas, H3 K27M-wildtype of midline structures

**DOI:** 10.1371/journal.pone.0249647

**Published:** 2021-08-04

**Authors:** Rouzbeh Banan, Arash Akbarian, Majid Samii, Amir Samii, Helmut Bertalanffy, Ulrich Lehmann, Christian Hartmann, Roland Brüning

**Affiliations:** 1 Department of Neuropathology, University of Heidelberg, Heidelberg, Germany; 2 Department of Neuropathology, Institute of Pathology, Hannover Medical School, Hannover, Germany; 3 Department of Neuroradiology, INI-Hannover, Hannover, Germany; 4 Department of Neurosurgery, INI-Hannover, Hannover, Germany; 5 Institute of Pathology, Hannover Medical School, Hannover, Germany; 6 Radiology and Neuroradiology, Asklepios Klinik Barmbek, Hamburg, Germany; Goethe University Hospital Frankfurt, GERMANY

## Abstract

**Purpose:**

The entity ‘diffuse midline glioma, H3 K27M-mutant (DMG)’ was introduced in the revised 4^th^ edition of the 2016 WHO classification of brain tumors. However, there are only a few reports on magnetic resonance imaging (MRI) of these tumors. Thus, we conducted a retrospective survey focused on MRI features of DMG compared to midline glioblastomas H3 K27M-wildtype (mGBM-H3wt).

**Methods:**

We identified 24 DMG cases and 19 mGBM-H3wt patients as controls. After being retrospectively evaluated for microscopic evidence of microvascular proliferations (MVP) and tumor necrosis by two experienced neuropathologists to identify the defining histological criteria of mGBM-H3wt, the samples were further analyzed by two experienced readers regarding imaging features such as shape, peritumoral edema and contrast enhancement.

**Results:**

The DMG were found in the thalamus in 37.5% of cases (controls 63%), in the brainstem in 50% (vs. 32%) and spinal cord in 12.5% (vs. 5%). In MRI and considering MVP, DMG were found to be by far less likely to develop peritumoral edema (OR: 0.13; 95%-CL: 0.02–0.62) (*p* = 0.010). They, similarly, were associated with a significantly lower probability of developing strong contrast enhancement compared to mGBM-H3wt (OR: 0.10; 95%-CL: 0.02–0.47) (*P* = 0.003).

**Conclusion:**

Despite having highly variable imaging features, DMG exhibited markedly less edema and lower contrast enhancement in MRI compared to mGBM-H3wt. Of these features, the enhancement level was associated with evidence of MVP.

## Introduction

Diffuse intrinsic pontine gliomas (DIPG) are the most common cause of brain tumor-related death in children [1]. Many of them demonstrate mutations of the genes *H3F3A* (histone H3.3), *HIST1H3B*, *HIST1H3C* (H3.1) and *HIST2H3C* (H3.2) encoding the H3 histones replacing lysine with methionine at position 27 (K27M) in the vast majority of the cases. Thereby, histones lose partially their functionality and the tumors undergo epigenetic alterations [2–6]. Since tumors harboring H3 K27M mutations were also frequently identified in the spinal cord, brainstem and diencephalon, and the patients showed a poor prognosis regardless of the tumor’s morphological appearance [1,7–11], they were defined as a new entity called ‘diffuse midline gliomas, H3-K27M-mutant’ (DMG) in the revised 4^th^ edition of the WHO classification of brain tumors of 2016 [12]. According to the criteria of the WHO classification of brain tumors, glioblastomas (GBM) are defined as malignant astrocytomas with necrosis and/or microvascular proliferation (MVP) [12]. Due to the poor prognosis of patients, GBM are also assigned a grade IV by the WHO classification [12]. Most DMG also show these histopathological characteristics and had thus been diagnosed as GBM before the identification of H3 K27M mutation. However, the definition criteria of the WHO classification for DMG do not enforce the detection of necrosis and/or vascular proliferation, so that a minor group of DMG can histopathologically manifest as a lower-graded glioma without considering H3 K27M mutations [12].

Although neurosurgical techniques to perform biopsies in the brainstem region are increasingly associated with lower morbidity [13], biopsy or resection is still often not possible due to the location of the lesion. Instead, the diagnosis of a DIPG in such a scenario is solely based on clinical and neuroradiological findings leading to aggressive radiotherapy as treatment option [14–16]. Previous trials of an additional temozolomide-based chemotherapy in patients with histologically diagnosed DIPG failed [17,18]. This could be explained by absence of O6-methylguanine-DNA-methyltransferase (*MGMT*) promoter methylation in DMG [8]. However, whether malignant midline gliomas without H3-K27M mutations might respond to temozolomide therapy remains speculative.

Since DIPG is usually detected and localized by imaging methods such as MRI, it seems important to identify a neuroradiological signature of DMG compared to other malignant midline gliomas that, in turn, may also contribute to biopsy guidance or other means of a preoperative assessment. For this, we retrospectively analyzed MRI characteristics of DMG compared to GBM of midline structures not carrying *H3F3A* or *HIST1H3B* mutations (mGBM-H3wt) as control group. In contrast to GBM, DMG does not necessarily have to histologically display necrosis and/or MVP and these two histopathological features show morphological similarities in the MRI signature. Moreover, tumor microvessel density has been shown to correlate with contrast enhancement parameters in gliomas [19,20] and is assumed to be associated with tumor-related edema through an increased vascular permeability that is due to abnormal vascular structure especially in high-grade gliomas [21,22]. According to these data, we have also analyzed MVP with respect to MRI features in DMG.

## Materials and methods

Tumor samples of diffusely infiltrating gliomas located in the brainstem, spinal cord, cerebellum, thalamus and other midline structures were retrospectively enrolled in this study. All patients had undergone open surgical intervention or stereotactic surgery in the International Neuroscience Institute (INI-Hannover) between 2004 and 2018. Sex, age and data regarding corticosteroid therapy at time of imaging due to its anti-edema effect were obtained from the patients’ files. Patients at the time of evaluation were primarily diagnosed and are therefore (apart from the neurosurgical intervention) to be considered as therapy-naive patients. The trial was approved by the Ethics Committee of the Hannover Medical University (MHH) (6960–2015). Patients or their next to kin gave written consent regarding inclusion into research studies. The study followed the declaration of Helsinki of 1964.

Formalin-fixed paraffin-embedded (FFPE) tissue blocks were retrieved from the archives of the Department of Neuropathology of the MHH. Hematoxylin and eosin-stained sections of the FFPE blocks were reviewed by two experienced neuropathologists (RBa, HA) [12,23]. Microvascular proliferations (MVP) and/or necrosis (geographical or palisading) in the context of diffuse malignant astrocytic histology were criteria for the diagnosis of glioblastoma (GBM) [12]. After DNA extraction we first analyzed our samples regarding *H3F3A* codon 27 status. The wildtype cases were then subject to *HIST1H3B* (codon 27) sequencing by pyrosequencing as described elsewhere [24].

**Fig 1:**
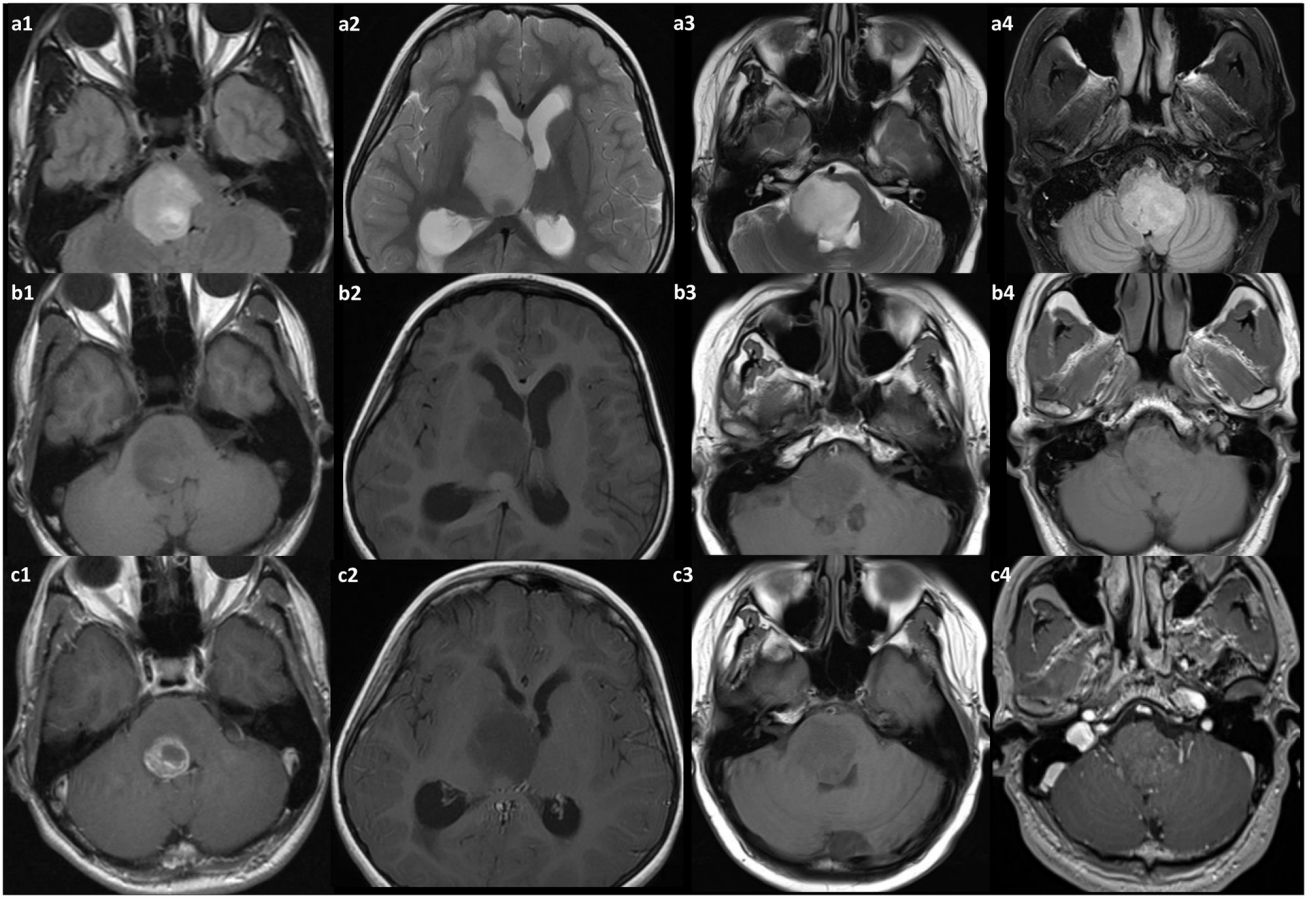
The spectrum of disease in the diffuse midline glioma patients with K27M mutation in MRI. In the upper row (a1, a2, a3, a4) examples of mass verification with T2-weighted FLAIR imaging are shown. In the middle row (b1, b2, b3, b4) the same cases with unenhanced T1-weighted imaging can be seen, and in the lower row (c1, c2, c3, c4) these patients are presented with T1-weighted imaging following contrast material administration—exhibiting the spectrum of contrast enhancement patterns from almost absent to rim enhancing. However, as a possible hallmark—edema in this entity is frequently absent.

For the retrospective imaging review, patients were included that matched following inclusion criteria: Imaging had to be digitally available on the local PACS system (SECTRA IDS 7, Sectra, Linköping, Sweden) (either 1,5T or 3T scanner), as unenhanced T1- and T2-weighted imaging, including at least one contrast-enhanced sequence, acquired approximately three minutes after injection. Sufficient image quality, as judged by the two readers, was required. For the retrospective evaluation, two readers with neuroradiological experience for 18 years (RBr) and 7 years (AA) read the image data on the above PACS system while blinded to each other’s results regarding the following aspects: 1.- Location (thalamus, brainstem, vermis, spinal cord, other), 2.- Absence/presence of necrosis, 3.- Contrast enhancement intensity (strong, intermediate, low or absent), 4.- Absence or presence of peritumoral edema (for examples please refer to Fig 1 upper row (a1, a2, a3, a4)) 5.- Absence/presence of mass effect, 6.- Absence/presence of rim enhancement and 7.- Singular lesion/multifocal lesions. Agreement or disagreement of the readers was noted. In case of different interpretations, a consensus discussion led to an integrated interpretation.

For statistical analysis, the software’s R Core Team 2019 (R Foundation; https://www.r-project.org/) and SPSS version 25 (IBM Corp., Armonk, NY, USA) were used. These tests were applied: Fisher’s exact test to analyze differences of groups in relation to variables and features included in our study, *t*-test to evaluate association of age and tumor type, logistic regression models using odds ratios (OR) and 95%-confidence interval (95%-CI). All (*p*) values reported are 2-sided and considered significant if < 0.05 throughout the whole analyses.

## Results

### DMG group

In total, 92 patients with diffusely infiltrating midline gliomas and without supratentorial tumors in their history were identified. The *H3F3A* K27M mutation was found in 35 tumors (38%) leading to a DMG diagnosis. In 57 Tumors (62%), no H3 K27M mutations in *H3F3A* or *HIST1H3B* were identified. Histopathological evaluations led to the diagnosis of mGBM-H3wt in 24/57 tumors (42%). In the DMG group, a total of 24/35 patients met the inclusion criteria and were included in analysis (mean age 27.0 years, standard deviation (SD) 13.8 years, from 5 to 54 years) consisting of 13 males and 11 females. Eleven patients were excluded from further analysis as imaging was not complete, insufficient or unavailable. Nine patients had undergone steroid therapy at time of imaging (9/24, 38%), while 13/24 patients received no treatment (54%). For 2/24 patients, no data regarding steroid therapy were available (8%). Histopathologically, 15/24 tumors showed MVP (62%) and 12/24 cases had necrosis (50%) (Table 1). Nine of the 24 DMG lesions were located in the thalamus (37.5%), 12/24 in the brainstem (50%), and 3/24 lesions were primarily located in the spinal cord (12.5%). Analysis of contrast-enhanced MRI showed necrosis in 17/24 patients (71%), while in 7/24 cases (29%), no necrosis was detected. Using subjective evaluation of both readers, there was strong contrast enhancement in 3/24 tumors (12.5%), intermediate in 9/24 cases (37.5%) and intermediate to low in 12/24 cases (50%) (here, a readers disagreement was on 2 cases: in one case enhancement was rated high by one reader and intermediate by the other; and in the other case it was rated low by one reader and absent by the other; as consensus both were rated at higher enhancement level). There was no lesion with a clear lack of contrast enhancement. Pronounced rim enhancement was observed in 10/24 cases (42%), while the remaining 14 cases had heterogeneous contrast enhancement but not focused on margins of the lesion. Regarding adjacent tissue, as judged by T2-weighted MRI, there were only 6/24 cases with detectable tumor-surrounding edema (25%), whereas 18/24 of the lesions had no detectable edema (75%). There were no disagreements between readers. In 7/24 patients, signs of intratumoral hemorrhagic signal changes were detected (using T1- or T2* weighted sequences), while in 17 patients (71%) these signs were absent. All lesions exhibited a mass effect (24/24), and all were within a single location (24/24) (Table 2). Typical examples of MRI findings are depicted in Fig 1.

**Table 1:** Patients’ characteristics including demography, genetic and histological findings, steroid therapy at the time of imaging and analysis of these features in relation to tumor type.

	DMG *n* (%)	mGBM-H3wt *n* (%)	Total	Association
Number of patients	24	19	43	
Sex				*P* = 1.000*
Male	13/24 (54)	9/19 (47)	22	
Female	11/24 (46)	10/19 (53)	21	
Age				*p* < 0.001**
Range	5–54	17–68		**95%-CI: 10.43–27.14**
Mean ± SD	27 ± 13.8	45.8 ± 13.0		
Steroid therapy	9/24 (37.5)	13/19 (68)	22	*P* = 0.078*
Histology				
MVP	15/24 (62)	19/19 (100)	34	*p* = 0.002*
Tumor necrosis	12/24 (50)	17/19 (89)	29	*p* = 0.009*

* Using Fisher’s exact test;

** Using *t*-Test.

DMG: Diffuse midline glioma, H3 K27M-mutant; mGBM-H3wt: Glioblastoma of the midline structures without *H3F3A/HIST1H3B* mutations, SD: Standard deviation; CI: Confidence interval; MVP: Microvascular proliferations.

**Table 2:** Summary of imaging findings and analysis of their difference in relation to tumor type using Fisher’s exact test.

Imaging findings		DMG *n* (%)	mGBM-H3wt *n* (%)	*p*-value
Location	thalamus	9/24 (37.5)	0.129	0.095
	brainstem	12/24 (50)	0.351	0.224
	spinal cord	3/24 (12.5)	0.618	0.417
Edema present		6/24 (25)	15/19 (79)	**0.001**
Rim enhancement		10/24 (42)	13/19 (68)	0.125
Tumor necrosis		17/24 (71)	17/19 (89)	0.257
Strong enhancement		3/24 (12.5)	11/19 (58)	**0.003**
Low enhancement		12/24 (50)	3/19 (16)	**0.026**
Mass effect		24/24 (100)	19/19 (100)	
Multifocal lesions		0	3/19 (16)	

DMG: Diffuse midline glioma, H3 K27M-mutant; mGBM-H3wt: Glioblastoma of the midline structures without *H3F3A/HIST1H3B* mutations.

### mGBM-H3wt group

In total, 19/25 patients with the histopathological diagnosis of mGBM-H3wt met neuroradiological inclusion criteria and were further analyzed in this group (mean 45.8 years, SD 13.0 years; 9 males and 10 females). Due to incomplete imaging, six patients had to be excluded from analysis. Steroid therapy was performed for 13/19 patients at time of imaging (68%), while no treatment was performed for six patients (32%). Microscopic MVP were found in all 19 tumors (100%) and necrosis was evident in 17 cases (89%). In 12/19 patients, the lesion was located in the thalamus (63%), 6/19 tumors were located in the brainstem (32%) and 1/19 had a spinal lesion (5%). MRI analysis followed by contrast enhancement revealed necrosis in 17/19 patients (89%). Contrast enhancement was rated strong in 11/19 patients (58%), intermediate in 5/19 (26%) and low in 3/19 cases (16%). There were no differences in readers’ evaluations. Rim enhancement was observed in 13/19 patients (68%). Peritumoral edema was found in 15/19 cases (79%). Mass effects were seen in all patients, and no intracranial metastasis was seen in the control group. Multifocal lesions were seen in 3/19 patients (16%) (Table 2).

Patients with DMG were significantly younger than those with mGBM-H3wt (*t*-test; *p* = 0.001, 95%-CI: 10.43–27.14). Using Fisher’s exact test, no association was found between sex or tumor location with tumor type. Histopathological evaluation revealed more frequent MVP (*p* = 0.002) and necrosis (*p* = 0.009) among mGBM-H3wt than DMG. Comparison of MRI features of both groups demonstrated significantly less frequent peritumoral edema in DMG (*p* = 0.001). Considering Steroid therapy at time of imaging that might potentially influence edema outcome, only 25% of DMG showed edema despite a low ratio of this therapy among patients (38%), and in the mGBM-H3wt cohort, this feature was observed in the vast majority of tumors (79%), while the rate of steroid therapy was relatively high (68%). In line with these frequencies, no association was found between steroid therapy and peritumoral edema both in DMG and mGBM-H3wt cohorts and among all patients regardless of tumor type. Moreover, mGBM-H3wt were associated with strong contrast enhancement (*p* = 0.003), whereas DMG more frequently showed low enhancement (*p* = 0.026). No association was found between tumor type and rim enhancement or necrosis (Table 2).

In the DMG group rim enhancement was observed in 10/15 tumors with MVP (67%) but in none of those lacking this feature (0/9) (*p* = 0.002). Two of 15 cases with MVP (13%) and only 1/9 tumor without MVP (11%) showed strong contrast enhancement and this difference was not significant. In contrast, we found a significantly higher frequency of intermediate or high enhancement level in tumors with MVP (11/15, 73%) compared to those without MVP (1/9, 11%) (*p* = 0.009). No association was found between MVP and peritumoral edema with a rate of 5/15 tumors with MVP showing edema (33%) and 1/9 tumor (11%) without MVP but edema in MRI. Necrosis in MRI was observed in 11/12 tumors with microscopic evidence of necrosis (92%) and 6/12 tumors without this feature (50%) and the difference was not significant. Due to limited number of mGBM-H3wt lacking necrosis (2 cases), and evidence of MVP in all cases, similar analyses could not be performed on the control group. We found, however, a threefold risk of having rim enhancement in this group (odds ratio 3.03) (Pearson’s Chi-squared test with Yates’ continuity correction).

The analysis of peritumoral edema and strong contrast enhancement using logistic regression models was limited due to the number of cases with these features making us include only two and one variables respectively. As for edema, we considered tumor type and MVP due to its association with this feature in Fisher’s exact test among all tumors included and its assumed role in developing tumor-related edema in high-grade gliomas as earlier described. According to a lack of association with edema in Fisher’s exact test in our both cohorts, we refrained from steroid therapy at time of imaging in the multivariate analysis. As a result, the tumor type of DMG was found to harbor the lowest risk of developing peritumoral edema (OR: 0.13; 95%-CL: 0.02–0.62) and this effect was significant (*p* = 0.010), whereas MVP was not found to be a predictor of edema independent of the tumor type (OR: 4.00; 95%-CL: 0.38–41.51) (*P* = 0.246). Regarding strong contrast enhancement, we solely analyzed tumor type making regression analysis actually serve as a further extension of the Fisher’s exact test described above. The analysis, accordingly, revealed DMG to have a significantly low risk of developing strong contrast enhancement (OR: 0.10; 95%-CL: 0.02–0.47) (*P* = 0.003) (Table 3).

**Table 3:** Analysis of peritumoral edema and strong contrast enhancement using logistic regression models.

Variables		*n* (% with edema/strong contrast enhancement)	Odds ratio	95%-confidence interval	*p*-value
*Peritumoral edema*					
Tumor type	mGBM-H3wt	15/19 (79)	REF.		
	**DMG**	**6/24 (25)**	**0.13**	**[0.02–0.62]**	**0.010**
MVP	No	1/9 (11)	REF.		
	Yes	20/34 (59)	4.00	[0.38–41.51]	0.246
*Strong Contrast enhancement*					
Tumor type	mGBM-H3wt	10/19 (53)	REF.		
	**DMG**	**3/24 (12.5)**	**0.10**	**[0.02–0.47]**	**0.003**

DMG: Diffuse midline glioma, H3 K27M-mutant; mGBM-H3wt: Glioblastoma of the midline structures without H3F3A/HIST1H3B mutations; REF.: Reference group; MVP: Microvascular proliferations.

## Discussion

DIPG are malignant, diffusely infiltrating gliomas of the brainstem, especially of the pons [12]. They often show histopathological characteristics of supratentorial GBM including microvascular proliferations and/or necrosis in context of a malignant astrocytic phenotype [1]. Other DIPG appear histopathologically as diffuse or anaplastic astrocytomas. In the 4^th^ edition of the 2007 WHO classification of brain tumors, DIPG was not defined as separate entity. Instead, it was recommended to classify and grade DIPG according to the definition criteria of supratentorial diffuse gliomas including diffuse astrocytomas WHO grade II & III and GBM [25]. The identification of *H3F3A* K27M mutations in a high number of DIPG in association with a generally observed malignant clinical course in patients independent of histopathological appearance [1,7–11] led to the definition of ‘Diffuse midline glioma, H3 K27M mutant’ (DMG) as a new entity in the revised 4^th^ edition of the 2016 WHO classification [12]. DMG were defined as diffuse glioma of midline structures with a predominant astrocytic phenotype and H3 K27M mutation. Regardless of supratentorial grading criteria of diffuse gliomas, the WHO classification assigns a WHO grade IV to DMG. After case reports documented that other brain tumor entities (e.g. pilocytic astrocytomas [26], ependymomas [27], gangliogliomas [28,29]) can also sporadically exhibit an H3 K27M mutation, the definition of DMG was clarified by cIMPACT-NOW Update 2, by excluding these entities [23]. DIPG without H3 K27M mutations are still classified and graded purely histopathologically. Such DIPG H3 K27M-wildtype have different genetic characteristics [30–34]. Since localization often makes a biopsy or resection impractical, diagnosis in absence of histopathological and molecular procedures must be carried out solely by neuroradiological examinations. Here we sought to determine MR imaging features in a selected patient cohort based on a brain tumor database in our center: while imaging features varied, particularly absence of peritumoral edema and low frequency of strong contrast enhancement seem to be an imaging indicator of DMG.

As expected, DMG were mostly found in the brainstem, but also in the thalamus and rarely spinal cord, whereas the mGBM-H3wt in our cohort were predominantly located in the thalamus, and less often spinal cord. These locations for DMG agree very well with those previously published [7,35]. However, we did not observe tumor in other midline sites such as third ventricle, pineal region, hypothalamus or cerebellum as previously described [7,36,37]. We, moreover, did not identify diffuse midline gliomas arising from the cerebellar hemispheres [24].

In our study, peritumoral edema, contrast enhancement ≥ intermediate and rim enhancement were associated with microscopically detected MVP among DMG, thereby suggesting a WHO grade IV in case of a DIPG. However, in this tumor group, no association was found between edema and MVP or between necrosis in MRI and that in histology. Hence, lack of edema and necrosis in imaging should not lead to the inadvertent downgrading of a potentially malignant tumor and their misinterpretation as signs of a lower WHO grade. Interestingly, the lack of tumor necrosis in diffuse gliomas in general was also recently described being a potential reason for misdiagnosis of these tumors [38]. Necrosis in MRI was observed in our DMG cohort in the majority of cases, however less frequently than in the mGBM-H3wt group (71% vs. 89%) despite statistically insignificant differences. This heterogeneous evidence of necrosis in DMG was reported in a pediatric population [39]. GBM are known to suffer a breakdown of the blood-brain barrier, frequently leading to increased enhancement of injected contrast medium. Accordingly, we found significantly strong or intermediate enhancement in 84% of our mGBM-H3wt, while only 50% of our DMG showed intermediate or strong enhancement with a high variability. Considering strong enhancement only, the difference between DMG and mGBM-H3wt was also significant. Thus, we were able to reproduce the published highly variable contrast enhancement of DMG initially in a pediatric population [39]. In contrast, in a large group, Schreck et al. found no significance with regard to frequency of contrast enhancement in DMG [40]. They, however, included H3 K27M-wildtype tumors in their control group comprising all diffuse midline gliomas of WHO grade II, III and IV that could potentially be responsible for this insignificance. Rim enhancement was observed in every third case of DMG (42%), almost similar to the rate reported (44%) [10], i.e. less frequent than in mGBM-H3wt group, yet statistically not significant. DMG had decreased odds to develop peritumoral edema (25% vs. 79% among controls) and this effect was independent of MVP. In line with our data, Aboian et al. reported only 4/24 pediatric patients with edema in their DMG group [39], Chiang et. al. named well-defined margins a typical feature [41]. They, however, described no significant difference of the MRI features analyzed between their DMG and controls located in posterior fossa despite suffering a statistically limited comparison due to their low control number (9 patients vs. 19 in our study). Moreover, they also considered H3 K27M-wildtype tumors in general as controls that could serve as a reason for this insignificant difference in addition to their statistical limitations.

In contrast to [39], our DMG cohort comprised rather adult patients (mean age 27.0 vs. 9.0 years). Presumably, referrals of the DMG patients in the INI-Hannover were responsible for the rather adult cases. Schreck et al., similarly, analyzed adult DMG patients (median 45.1 years), yet considering exclusively contrast enhancement in the MRI analysis of their DMG [40].

In summary, no single neuroradiological criterion for differentiation between DMG and mGBM-H3wt could be established. However, it was striking that DMG showed significantly less edema and strong contrast enhancement as well as more frequent low enhancement levels than mGBM-H3wt. All these characteristics are attributed to a disturbed blood brain barrier in the context of malignant gliomas, and MVP is usually referred as cause of this disturbance [42,43]. Owing to the observation that such a barrier disorder is much less common in DMG than in mGBM-H3wt, and given the definition criteria of DMG by the 2016 WHO classification that MVP does not necessarily have to be present, it may be feasible that such a discrepancy in these imaging features is related to differences between DMG and mGBM-H3wt in terms of their vasculature, i.e. absence of MVP in those DMG lacking edema and/or showing low contrast enhancement. In favor of such an argument, we found both a significantly lower frequency of MVP in our DMG cases compared to our mGBM-H3wt group, and an association of MVP absence with low enhancement level in DMG. Nevertheless, no association between MVP and peritumoral edema was observed. Furthermore, it seems conceivable that due to DMG biology, intratumoral vasculature shows less severe loss of function than in mGBM-H3wt. Systematic analyses of DMG vasculature in comparison to that of mGBM-H3wt have not yet been published.

However, the finding of less frequent edema as well as the frequency of other findings may assist as features that can be used in the future to classify brain tumors and be part of more advanced image analysis algorithms [44]. It remains an open question whether machine-learning algorithms can support MR image analysis in this context. Interestingly, initial reports on gliomas indicate high accuracy [45,46]. Recently, a lower value of apparent diffusion coefficient (ADC) in peritumoral regions and an abnormal ADC ratio was published [47].

Limitations of the present study include the relatively small number of patients not enabling us to perform comprehensive multivariate analyses considering multiple variables, the single center approach, further group differences such as age and gender, previous neuro-oncological treatments, and the nature of its retrospective evaluation. Moreover, for most of the patients, no complete examination of the spinal axis was available; thus, aspects of spinal metastasis could not completely be clarified by evaluation. Furthermore, the rate of 38% of patients with diffuse midline glioma harboring H3F3A K27M mutation in our cohort is below the reported frequency of 50–80% [23]. However, these published data refer predominantly to pediatric cohorts, whereas currently no epidemiological data are available regarding the proportion of DMG patients in mixed pediatric/adult cohorts [48]. While the reported median age of patients with DMG is 5–11 years [12], the median age of the patients presented here is 27 years. Thus, it is to assume that the frequency of patients diagnosed with DMG decreases noticeably when mixed groups are analyzed.

In conclusion, DMG in our study cohort had highly variable features on MRI, but exhibited significantly less edema and lower contrast enhancement in MRI compared to the mGBM-H3wt control group. Biopsy and neuropathological analysis of diffuse midline gliomas remains the diagnostic gold standard for further treatment planning. For instance, we have recently demonstrated that patients with infratentorial diffuse astrocytomas harboring IDH mutation have a better prognosis than those with DMG [49]. Furthermore, single studies suggest that patients with infratentorial gliomas and a combined H3F3A K27M and BRAF V600E mutation have a better prognosis [24], while those with tumors of such a localization and combined H3 K27M and IDH mutations are more likely to be prognostically similar to DMG patients [49]. However, since bioptic sampling of diffuse infratentorial gliomas is often not possible for individual or institutional reasons, neuroradiological diagnostics remains the only option for differentiation of tumor entities for therapy planning. In order to derive increasingly unambiguous neuroradiological criteria for the differentiation of such subgroups of diffuse gliomas, further studies are required. A molecular differentiation of the tumors of the central nervous system beyond single biomarkers is now available using comprehensive epigenetic analyses [50]. An association of neuroradiological and epigenetic signatures of infratentorial diffuse gliomas is also of particular interest and should be analysed in future.
